# OD08 Health Technology Assessment Timelines And Outcomes Of Common Compounds In France, Germany, Sweden, And Poland From 2014 To 2022

**DOI:** 10.1017/S0266462324001442

**Published:** 2025-01-07

**Authors:** Belen Sola-Barrado, Tina Wang, Neil McAuslane

## Abstract

**Introduction:**

The Regulation (EU) 2021/2282 on health technology assessment (HTAR) takes effect in January 2025 and aims to improve and accelerate patients’ access to new health technologies. We examine HTA timelines and outcomes for new active substances (NASs) commonly appraised in all of France, Germany, Poland, and Sweden from 2014 to 2022, providing insights into the European landscape before HTAR.

**Methods:**

Public data was extracted from the HTA reports from the corresponding health authorities from France (HAS), Germany (IQWiG), Poland (AOTMiT), and Sweden (TLV) published between 1 January 2014 to 31 December 2022. NASs appraised by HTA in the four jurisdictions were referred to as common compounds. Time to recommendation was calculated as European Medicines Agency approval to HTA recommendation in the corresponding country. Differences in time parameters were assessed using the Kruskal–Wallis test. HTA recommendations were categorized as positive, positive with restrictions, and negative.

**Results:**

A total of 97 common compounds (75% chemical entities and 25% biotechnology products) were identified (388 HTA appraisals). Notably, 47/97 (48%) NASs were associated with anticancer drugs (ATC-code=L). The median (interquartile range) time to recommendation differed by jurisdiction: 128 (113, 169) days in Germany; 203 (151, 308) days in Sweden; France, 209 (162, 294) days; Poland, 479 (330, 738) days (p-value <2.2e-16). In addition, 62/97 (63.9%) products received their initial HTA recommendation in Germany, followed by 25/97 (25.8%) products in Sweden, and 10/97 (10.3%) in France. Only 7/97 (7%) of products exhibited unanimous HTA outcomes across jurisdictions.

**Conclusions:**

Discrepancies in the time to HTA recommendation were identified across four prominent European jurisdictions, and consensus in HTA outcomes was notably limited across countries, underscoring the intricate landscape preceding the enforcement of the HTAR. The future implementation of HTAR holds the potential to address these disparities, fostering greater harmonization in HTA processes and outcomes across the European healthcare landscape.

